# Revision of *Coelastrella* (Scenedesmaceae, Chlorophyta) and first register of this green coccoid microalga for continental Norway

**DOI:** 10.1007/s11274-020-02897-0

**Published:** 2020-09-11

**Authors:** F. Goecke, J. Noda, M. Paliocha, H. R. Gislerød

**Affiliations:** 1 Department of Plant Sciences, Faculty of Biosciences, Norwegian University of Life Sciences, P.O. Box 5003, 1432 Ås, Norway; 2 Laboratory of Photosynthesis, Centre Algatech, Institute of Microbiology, The Czech Academy of Sciences (CAS), Třeboň, Czech Republic

**Keywords:** 18S rDNA, ITS, Algae phylogeny, Fatty acids, Electron microscopy, Strain FGS-001

## Abstract

A terrestrial green microalga was isolated at Ås, in Akershus County, Norway. The strain corresponded to a coccoid chlorophyte. Morphological characteristics by light and electron microscopy, in conjunction with DNA amplification and sequencing of the 18 s rDNA gene and ITS sequences, were used to identify the microalgae. The characteristics agree with those of the genus *Coelastrella* defined by Chodat, and formed a sister group with the recently described *C. thermophila* var. *globulina*. *Coelastrella* is a relatively small numbered genus that has not been observed in continental Norway before; there are no previous cultures available in collections of Norwegian strains. Gas chromatography analyses of the FAME-derivatives showed a high percentage of polyunsaturated fatty acids (44–45%) especially linolenic acid (C18:3n3; 30–34%). After the stationary phase, the cultures were able to accumulate several carotenoids as neoxanthin, pheophytin a, astaxanthin, canthaxanthin, lutein, and violaxanthin. Due to the scarcity of visual characters suitable for diagnostic purposes and the lack of DNA sequence information, there is a high possibility that species of this genus have been neglected in local environmental studies, even though it showed interesting properties for algal biotechnology.

**Electronic supplementary material:**

The online version of this article (10.1007/s11274-020-02897-0) contains supplementary material, which is available to authorized users.

## Introduction

Chlorophyta are an ancient and taxonomically diverse lineage of green algae with approximately 8000 described species and an estimated of 5000 still undescribed species (Hadi et al. 2016). For a long time, the classification of these organisms has been entirely based on morphological and cytological features of vegetative stages in their life cycle (Darienko et al. 2015). Unfortunately, the identification of coccoid green algae often presents a challenge for algal taxonomists due to the scarcity of visual characters suitable for diagnostic purposes and the lack of DNA sequence information (Škaloud et al. 2016).

Traditionally, the order Chlorococcales sensu lato grouped the coccoid taxa and represented one of the most diverse groups of photoautotrophic cryptogams. However, later studies have managed to transfer many taxa to other orders or classes i.e. Chlorophyceae, Trebouxiophyceae, and Prasinophyceae (Krienitz and Bock 2012). Moreover, phenotypic plasticity and the presence of cryptic species have contributed to taxonomy complication, resulting in constant reassignments in these microalgae (Eliáš et al. 2010; Malavasi et al. 2016).

Members of the subfamily Coelastroideae have been previously placed in the families Oocystaceae, Chlorellaceae, and Scotielloideae based on shape, reproduction, cell wall morphology and composition (Kalina and PunČochářová 1987), until phylogenetic molecular studies were applied. Nowadays, DNA amplification and sequencing of the 18S rDNA and internal transcribed spacer (ITS) studies placed Coelastroideae within the family Scenedesmaceae, order Sphaeropleales (Hanagata 1998, 2001; Hegewald and Hanagata 2000, 2002; Hegewald et al. 2010; Kaufnerová and Eliáš 2013; Lee et al. 2016; Ancona-Canché et al. 2017). According to AlgaeBase (Guiry and Guiry 2020), the subfamily Coelastroideae comprises seven genera: *Coelastrella* Chodat 1922 (16 species flagged as accepted taxonomically), *Scotiellopsis* Vinatzer 1975 (1 sp.), *Asterarcys* Comas Gonzales 1981 (1 sp.), *Hariotina* Dangeard 1889 (2 spp.), *Dimorphococcus* Braun 1855 (3 spp.), *Coelastrum* Nägeli 1849 (30 accepted species), and *Graesiella* Kalina and PunČochářová (1987) (1 sp.).

The genus *Coelastrella* has been known for almost 100 years and it is relatively small numbered. *Coelastrella* spp. are coccoid, elliptical until citriform. They occur as unicellular microalgae or in few-celled aggregations. These species are peculiar by their sculptured cell wall with 16–40 meridional ribs with or without polar thickenings (Uzunov et al. 2008; Kaufnerová and Eliáš 2013). Ultrastructurally, cells are uninucleate, present numerous conspicuous vacuoles, a single cup-shaped and parietal chloroplast, each with one pyrenoid surrounded by 2(3) starch plates. The cell wall is double layered, with an inner cellulose component and an outer trilaminar one where acetolysis-resistant material (sporopollenin) resides (Tschaikner et al. 2007a, 2008). Asexual reproduction occurs by 2–16 autospores released by rupture of parental cell wall (Guiry and Guiry 2020). Together with other morphological features like cell form, chloroplast and pyrenoid structures, the characteristic differences in the wall sculptures are useful for identification of the species (Gärtner and Ingolić 1993; Hanagata et al. 1996; Tschaikner et al. 2007a, b). The type species of the genus is *Coelastrella striolata* Chodat 1922.

Recently, diverse studies have reported the biotechnological interest of this genus due to pigments and fatty acid content, as well as for a potential use for bioremediation (Abe et al. 2007; Hu et al. 2013; Dimitrova et al. 2017; Kawasaki et al. 2013; Luo et al. 2016; Thao et al. 2017; Wang et al. 2019b). In the last decades, the biotechnological use of microalgae has raised the interest from the industry (Wijffels et al. 2010). Microalgae have been found to be the most promising feedstock in terms of their biomass productivity, high oil content, strong adaptive capacity to adverse environments, heavy metals, toxicants, high CO_2_ concentration and no competition with cultivable land (Chisti 2007). The screening of native algal species and strains is necessary for assessing the biotechnological potential of microalgae, especially in harsh environments; some of those species may be more beneficial than the commercially available strains.

In the present study, we report a terrestrial coccoid green microalga FGS-001, isolated at Ås, in Akershus County, Norway, which was identified as a *Coelastrella* strain, until now not described for continental Norway. Furthermore, very little is known about the distribution of this genus worldwide. The objectives of the study were (1) to increase knowledge on terrestrial Norwegian microalgae, (2) to characterize the isolated strain, (3) to determine its pigment and fatty acid composition profile, (4) to determine the phylogenetic relationships with known algal strains.

## Material and methods

### Microorganism isolation, medium and culture conditions

The strain FGS-001 was isolated from a foliose, land-living colony of *Nostoc commune*, known locally as «*glye*» in Norwegian language. A sample of the cyanobacterium colony was collected in summer 2016 outside the Center for Climate Regulated Plant Research (SKP), Norwegian University of Life Sciences, Campus Ås, Akershus County, South East Norway (N 59° 40′ 5.81292′′, E 10° 46′ 14.92156′′ (EU89)). The sample was washed with sterile Milli-Q water, exposed to light and enriched nutrient solution (a modified Kristalon Indigo medium) without aeration in a 50 mL conical flask.

Initially, only cyanobacteria belonging to Nostocales were visible. After 3–4 weeks of culture, green microalgae were observed under light microscope, growing inside fragments of the colony. Further isolation of the microalgae was achieved by consecutive transfers to fresh medium.

Kristalon Indigo standard nutrient solution according to producer (Yara, Norway) is composed NO_3_^−^ 7.5%; NH^4+^ 1%; P 4.9%; K 24.7%; Mg 4.2%; S 5.7%; B 0.027%; Cu 0.004%; Fe 0.2%; Mn 0.06%; Mo 0.004%, and Zn 0.027%. A concentration was used of 0.01 g per liter of milli-Q water, enriched with 0.01 g urea and 0.002 g Opti-P 0-20-0 (Yara, Norway). The microalga was able to grow in a Tris–acetate–phosphate (TAP) (–) acetate media (see Harris 1989), and modified Provasoli nutrient media (West and McBride 1999), as well.

Finally, the isolated strain was able to grow in tubular photobioreactors (250 mL). It was cultivated under continuous illumination at a surface incident irradiance of 175 µmol m^2^/s at 20.0 ± 2 °C at constant aeration under filtered air flow containing 1 ± 0.2% CO_2_ (v/v), and at an initial pH of 7.0.

Dry matter was determined gravimetrically. Aliquots (2 mL) of samples were harvested from the photobioreactors in pre-weighed tubes by centrifugation. The supernatant was discarded, and pellets were dried at 105 °C until they reached a constant weight (Goecke et al. 2015). Growth curve was determined by linear regression of the natural log of cell biomass vs. time for the data plotted in Fig. 4.

A voucher specimen will be deposited in the Norwegian Culture Collection of Algae (NORCCA) (https://niva-cca.no/).

### Optical microscopy

To obtain a detailed morphological characterization of cultured microalgae, we investigated it by different microscopical techniques.

Light microscopy observations were performed using a Leica DM5000B microscope (Leica CTR5000, Leica Microsystems Limited, Heerbrugg, Switzerland) equipped with the attached Leica camera (DC200), and microphotographs were processed with the Leica Application Suite v4.3 image program (LAS 4.3).

Additionally, to corroborate that the cells are uninucleated, samples were stained for 15 min with the nuclear marker DAPI (4′,6-diamidino-2-phenylindole; 10 µg/mL stock; Sigma-Aldrich, Saint Louis Missouri, USA), and analyzed using a UV filter under fluorescence microscopy.

### Transmission electron microscopy (TEM)

Samples from two different culture ages (exponential and stationary phase) were fixed for transmission electron microscopy using chemical fixation protocols according to Olsen et al. (2015). Briefly, cells were harvested, centrifuged and prefixed in 1.25% glutaraldehyde and 2% formaldehyde in phosphate-buffered saline (PBS) solution for a minimum of 24 h. After washing several times in PBS and cacodylate buffer (0.1 M, pH 7.2), the cells were post fixed for 1 h at room temperature in 1% osmium tetroxide (OsO_4_), and washed again in cacodylate buffer. After dehydration through an ethanol series (15 min in 70, 90, 96%, and 4 × 15 min in 100%), cells were infiltrated and embedded in in LR-White resin (Electron Microscopy Sciences, USA). Ultrathin sections were prepared using a Leica Ultramicrotome EM UC7, counterstained with 4% aqueous uranyl acetate and 1% potassium permanganate (KMnO_4_) for 5 min and then washed in distilled water before examined and photographed in the transmission electron microscope (FEI Morgagni 268), using a Veleta CCD camera.

### Scanning electron microscopy (SEM)

Algae were treated using the same fixing method described above for TEM. After, coverslips coated with 1 mg mL^−1^ poly-l-lysine were placed to allow algae to settle for 20 min. The fixed algae culture for SEM examination were washed thoroughly in 0.1 M sodium cacodylate buffer (SCB) and dehydrated with 10 min steps in ascending ethanol series (50–100%) as in Wiik-Nielsen et al. (2016). The samples were processed in a BAL-TEC Critical Point Dryer CPD 030, (BAL-TEC AG Lichtenstein), and a thin conductive coating of gold–palladium was applied to the samples using a Polar on Sputter Coater SC7640 (Quorum Technologies, UK). The coated samples were mounted on aluminum stubs, examined and photographed with a Zeiss EVO-50-EP scanning electron microscope at an accelerating voltage of 20 kV in the secondary emission mode.

### DNA amplification and sequencing

For this study, the nuclear 18S rDNA gene and ITS regions of the alga were sequenced as described below.

Cells from 1 mL of algal culture were harvested by centrifugation, resuspended in 50 μL of EDTA 10 mM pH 8, and incubated at 100 °C for 10 min. Then, samples were cooled down at 4 °C for 10 min and resuspended by vortex. Finally, after 1 min of centrifugation at 10.000 rpm, 1 μL of supernatant was used to perform the PCR reactions.

The primer sets HET F (5′-ACCTGGTTGATCCTGCCAGTAGTCATAC-3′) and HET R (5′-GGTTCACCTACGGAAACCTTGTTACGACTTCA-3′) (Cavalier-Smith and Chao 2006), were used for the amplifications of the 18S rDNA regions.

The primer sets ITS1 (5′-TCCGTAGGTGAACCTGCGG-3′)/ITS4 (5′-TCCTCCGCTTATTGATATGC-3′) (White et al. 1990), were used for the amplifications of the ITS regions.

All fragments were amplified with Q5 High-Fidelity DNA Polymerase (New England BioLabs).

Purified and concentrated PCR products were used as template for Sanger dideoxy sequencing at GATC Services (Eurofins, Germany), using the same primers sets employed in the amplification of the fragments, and 18F2 (5′-GCTCGTAGTTGGATTTCTGG-3′) (this study), in the case of 18S rDNA regions.

New sequences generated by this investigation were submitted to the nucleotide database GenBank from the National Center for Biotechnology Information (NCBI), with GenBank accession numbers MK064224 and MK040329.

### Phylogenetic analysis

The identity of the isolates was assessed by a phylogenetic analysis using BEAST v1.10.4 (Suchard et al. 2018). Sequences for 18S rRNA and internal transcribed spacer (ITS) were taken from Wang et al. (2019b) and downloaded from NCBI’s GenBank (NCBI Resource Coordinators 2017) database using the respective accession numbers. Nucleotide sequences generated in this study were added to the data set, and a multiple sequence alignment was computed with MAFFT v7.310 using the L-INS-i strategy (Katoh et al. 2002; Katoh and Standley 2013). To remove uninformative sites and reduce the matrix to the loci of interest, the alignment was trimmed with trimAl (Capella-Gutiérrez et al. 2009) with the parameters -gt 0.8 -st 0.001 -cons 0.6. After manual inspection and adjustment of the alignment, the best nucleotide substitution model was determined based on AICc calculations by the modelTest function from the R package phangorn v2.5.3 (Schliep 2011; Darriba et al. 2012) using R v3.6.0 (R Core Team 2019). Thus, nucleotide substitutions in the 18S sequences were approximated with the GTR + Γ + I model (Hasegawa et al. 1985; Tavaré 1986; Yang 1994). For the ITS sequences, the GTR + Γ substitution model was used (Hasegawa et al. 1985; Tavaré 1986). Heterogeneity of the substitution rate was approximated by four discrete Γ categories in both cases. Phylogenetic trees were inferred assuming an uncorrelated relaxed molecular clock prior following a log-normal distribution (Drummond et al. 2006), assuming a Yule speciation process (Yule 1925; Gernhard 2008). Two separate Markov chain Monte Carlo (MCMC) analyses were run. Each run lasted 1.0 × 10^9^ generations and trees were sampled every 5000 generations. The output from both MCMC chains was combined using LogCombiner v1.10.4 (Suchard et al. 2018), and 25% of the trees discarded as burn-in. The tree with maximum clade credibility was inferred with TreeAnnotator v1.10.4 (Suchard et al. 2018), rescaled to resemble mean node height, and visualized with FigTree v1.4.4 (Rambaut 2018). *Chloromonas serbinowi* UTEX 492 and *Chlamydomonas reinhardtii* UTEX 90 were used as out group with enforced monophyly for the Sphaeropleales–Chlamydomonadales split.

### Analysis of pigment composition

Aliquots of 25–40 mg of freeze-dried algae material were weighed into lysis tubes (Type C, Analytik Jena, Jena, Germany) and 500 µL of ethanol (gradient grade, Merck, Darmstadt, Germany) were added. Cells were mechanically broken in a swing mill (MM 2000, Retsch, Haan, Germany) for 3 min. Afterwards the cells were centrifuged at 3000 rcf (5415R, Eppendorf, Hamburg, Germany) and the supernatant was recovered. This procedure was repeated twice until the supernatant was colorless. The combined extracts were dried under nitrogen at 40 °C in an evaporator (EVA-EC1 with metal block thermostat EC-1 V-130, both VLM, Bielefeld, Germany) and afterwards resuspended in a defined volume of ethanol and filtered through a 0.45 µm membrane filter (Chromafil Xtra PET -45/25, Macherey–Nagel, Düren, Germany). The samples were measured in an Ultra-performance liquid chromatography mass spectrometry (UPLC-MS), coupled to photodiode array detection (PDA), a UPLC-PDA-MS system (Waters, Milford, USA) with a Cortecs C18 collumn (2.7 µm, 90 Å, 3 × 100 mm, Waters, Milford, USA), with a gradient of Millipore water and acetonitrile (hypergrade for LC–MS, Merck, Darmstadt, Germany) both acidified with 0,01% formic acid (99% ULC/MS, Biosolve B.V., Valkenswaard, Netherlands). Starting conditions were 70% water, decreasing to 10% after 4 min. These conditions were kept stable further 10 min. Afterwards a washing step with 70% water was attached for 4 min. The flow velocity was 0.5 mL/min continuously. Column temperature was set at 40 °C and the spectra were measured via a PDA (2998 PDA Detector, Waters, Milford, USA) in a range of 200 to 800 nm. The mass spectrometer with electronspray ionization (ESI) (Acquity QDA, Waters, Milford, USA) was operated in positive mode with a cone voltage of 15 V and a probe temperature of 600 °C, measuring in a range of 150 to 1250 m/z.

As references standards Astaxanthin (≥ 97% from *Blakeslea trispora*, Sigma-Aldrich, St. Louis, USA) and Lutein (pharmaceutical secondary standard, Sigma-Aldrich, St. Louis, USA) were used. Further carotenoids were determined by comparison with literature.

For the photometric measurements (for full spectrum for chlorophyll detection), samples were prepared similarly in quantities of 5 to 10 mg, extracted in ethanol (≥ 99.5%, Ph.Eur., Carl Roth, Karlsruhe, Germany), but measured in different concentrations in a multiwellplate (96F, TPP Techno Plastic Products AG, Trasadingen, Swiss) in an microplate reader (Infinite M Plex, Tecan, Männedorf, Swiss) or alternatively in a photometer (DR 6000, Hach Lange, Düsseldorf, Germany) with a spectrum from 300 to 800 nm in steps of 2–5 nm. Due to biomass limitations, pigments were detected although not quantified.

### Analysis of fatty acid composition

Conversion and extraction of algal lipids to fatty acid methyl esters (FAME) was done by the method of O’Fallon et al. (2007) with a minor modification concerning the volumes. Samples (0.3 g of freeze-dried sample) were placed into Pyrex culture tubes to which 0.3 mL of the C13:0 internal standard (0.5 mg of C13:0/mL of MeOH), 0.56 mL of 10 M KOH in water, and 4.24 mL of MeOH were added. The tubes were incubated in a 55 °C water bath for 1.5 h with vigorous hand-shaking for 5 s every 20 min to properly permeate, dissolve, and hydrolyze the sample. After cooling below room temperature in a cold tap water bath, 0.46 mL of 24 M H_2_SO_4_ in water was added. The tube was mixed by inversion and with precipitated K_2_SO_4_ present was incubated again in a 55 °C water bath for 1.5 h with hand-shaking for 5 s every 20 min. After cooling, 2.4 mL of heptane was added, and the tube was vortex-mixed for 5 min. The tube was centrifuged for 5 min in a tabletop centrifuge, and the hexane layer, containing the FAME, was placed into a gas chromatography (GC) vial. The vial was capped and placed at − 20 °C until analysis.

The fatty acid composition of the FAME was determined by capillary GC on a RT-2560, 100 m × 0.25 mm × 0.20 µm capillary column (Restek) installed on a Trace GC ULTRA gas chromatograph equipped with a Triplus autosampler with PTV inlet and a flame ionization detector, and controlled by Chromeleon 7.2 Chromatography Data System (Dionex, ThermoFisher Scientific). The initial oven temperature was 140 °C, held for 5 min, subsequently increased to 240 °C at a rate of 4 °C/min, and then held for 20 min. Helium was used as the carrier gas at a flow rate of 2 mL/min, and the column head pressure was 270 kPa. Both the injector and the detector were set at 250 °C. The injector was set in split mode and the split ratio was 20:1. Fatty acids were identified by comparing their retention times with the fatty acid methyl standards (Supelco standard FAME mixture) described previously.

## Results

### Light microscopy

**Fig. 1:**
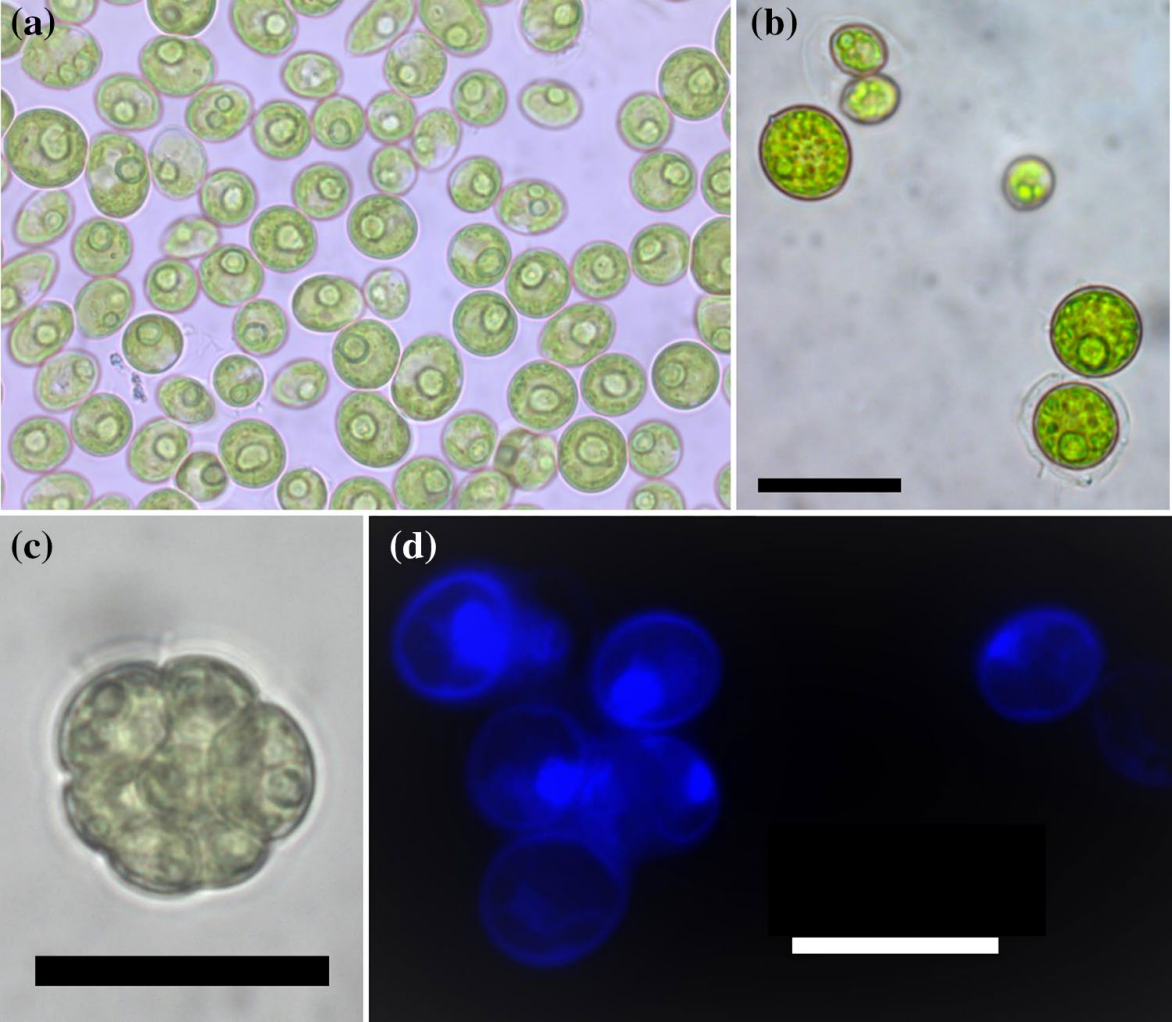
Unialgal culture of the strain FGS-001. **a** vegetative cells are green, unicellular and grows in variable forms from spheroidal to ellipsoidal. A single pyrenoid is visible; **b** cells with a smooth polar thickening were observed as well as others still surrounded by sporangium walls; **c** autospores in aggregation with a pyrenoid; **d** uninuclear cells were observed stained by DAPI under fluorescence. Scale bar: **a**–**c** = 20 µm; **d** = 10 µm

The isolated strain FGS-001 is a unicellular dark green microalga, although some cells were in aggregation. It grows in variable forms from globose or spheroidal to ellipsoidal (Fig. 1a). The cells presented variable sizes, but usually were (6–)7–10(–13) µm long (with an average of 8.42 ± 1.34 µm) and (4.5–)6–9(–11) µm wide (with an average of 7.14 ± 1.39 µm) (Fig. 1). Cell wall appears to be hyaline, and a cup-shaped chloroplast was easy to observe. A stricken and single pyrenoid was clearly visible in the vegetative cells as well as in autospores (Fig. 1a, b).

Meridional ribs were very difficult to observe under light microscopy. Although a smooth, apical thickening was visible in few cells (Fig. 1b).

Asexual reproduction takes place by autospores formed by successive bipartition of the protoplast to produce 4 to 16 spores within the mother cell (Fig. 1c). Autospores are elongated and polar thickenings were easier to notice, as well as their pyrenoids. They are discharged through rupture of the cell wall of the sporangium although some remain surrounded by sporangium walls (Fig. 1b, c). No sexual reproduction was observed.

### Electron microscopy.

**Fig. 2:**
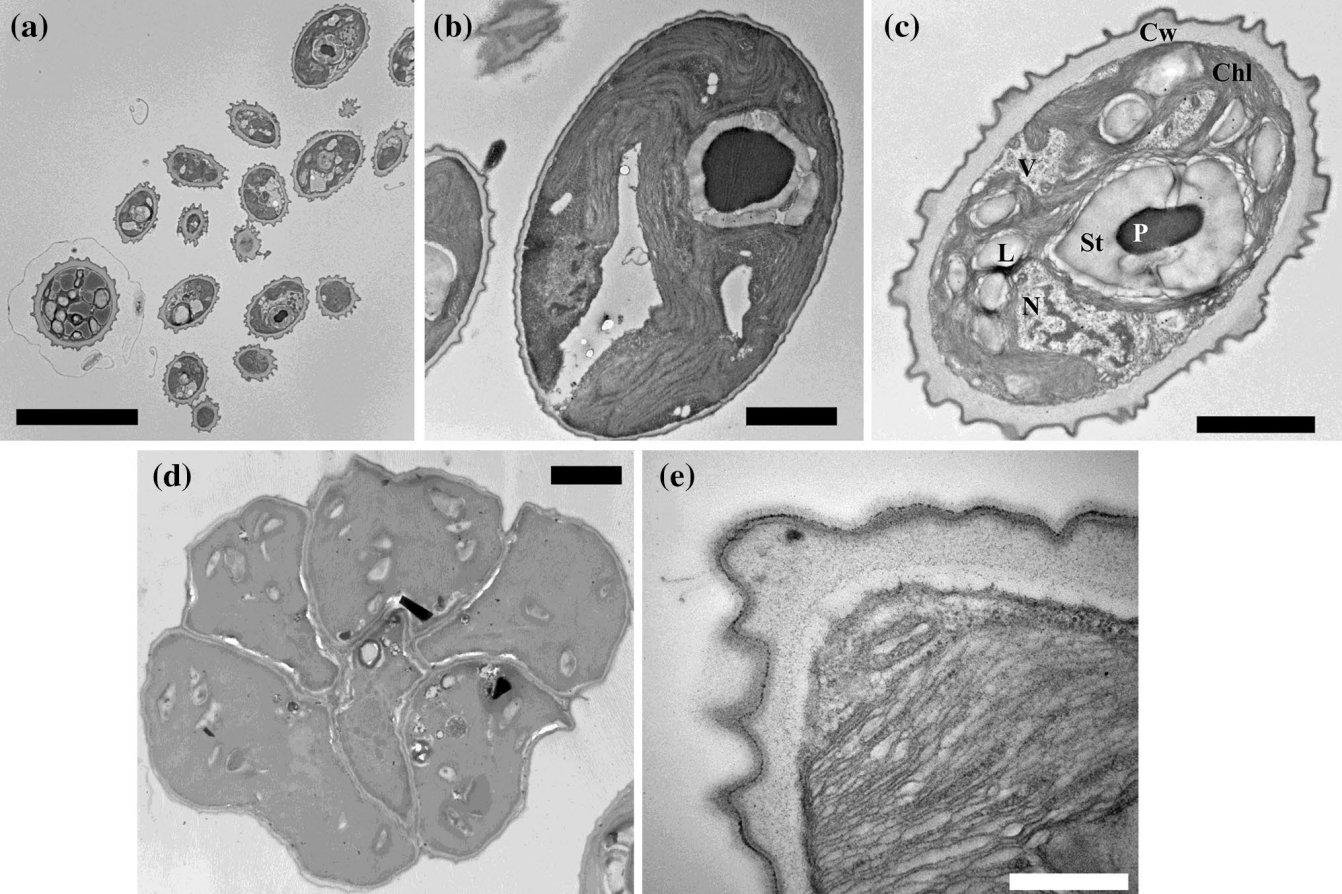
**a** Transmission electron microphotographs of a group of vegetative and reproductive cells of *Coelastrella* sp. FGS-001 isolated from Ås, Akershus, Norway. Scale bar of 10 µm. **b** Young vegetative cell at exponential growth. A cup-shaped chloroplast (Ch) with dense thylakoids, the associated pyrenoid (P) structure with starch plates, and a thin cell wall, are clearly visible on the strain. Very smooth ribs are also present at the cell wall surface. **c** Autospore showing the single nucleus (N), vacuoles (V), thick starch plates (St), lipid droplets (L) and a thick and irregular cell wall (CW), with clear ribs of different sizes. **d** Autosporangia showing the formation of autospores, six cells are visible. **e** Cell wall detail with an outer trilaminar component. Scale bar: **a** = 10 µm; **b**–**d** = 2 µm; and **e** = 0.5 µm

Transmission electron microphotographs of a group of vegetative and reproductive cells of *Coelastrella* sp. FGS-001 is shown in Fig. 2. Figure 2a is an example of the variable forms, from globose to ellipsoidal cells, which were found with variable sizes. The distribution of cell organelles is showed in detail by this technique. Vegetative cells presented a thin cell wall, where very smooth ribs were also visible at the cell wall surface (Fig. 2b). Internally, the single chloroplast proliferates throughout the cell with dense thylakoids; it presents a large cup-shaped form, which is associated with a single, prominent, spheroidal, and central pyrenoid structure with a ring of starch plates (n = 2) surrounding it, clearly visible (Fig. 2a–c). The single nucleus was located next to the pyrenoid (Fig. 2c). Occasionally, a second pyrenoid was observed by cell division (not shown).

The autospores presented a slightly smaller size than the vegetative cells, with a single nucleus, several vacuoles, lipid/starch droplets, thicker starch plates surrounding the pyrenoid, and a thick and irregular cell wall, with clear ribs of different sizes at the surface level (Fig. 2c). A variable number of autospores were visible at the autosporangia (Fig. 2d).

The cell wall of *Coelastrella* sp. FGS-001 consists of multiple layers, with an inner cellulose component and an outer apparently trilaminar one (Fig. 2e).

**Fig. 3:**
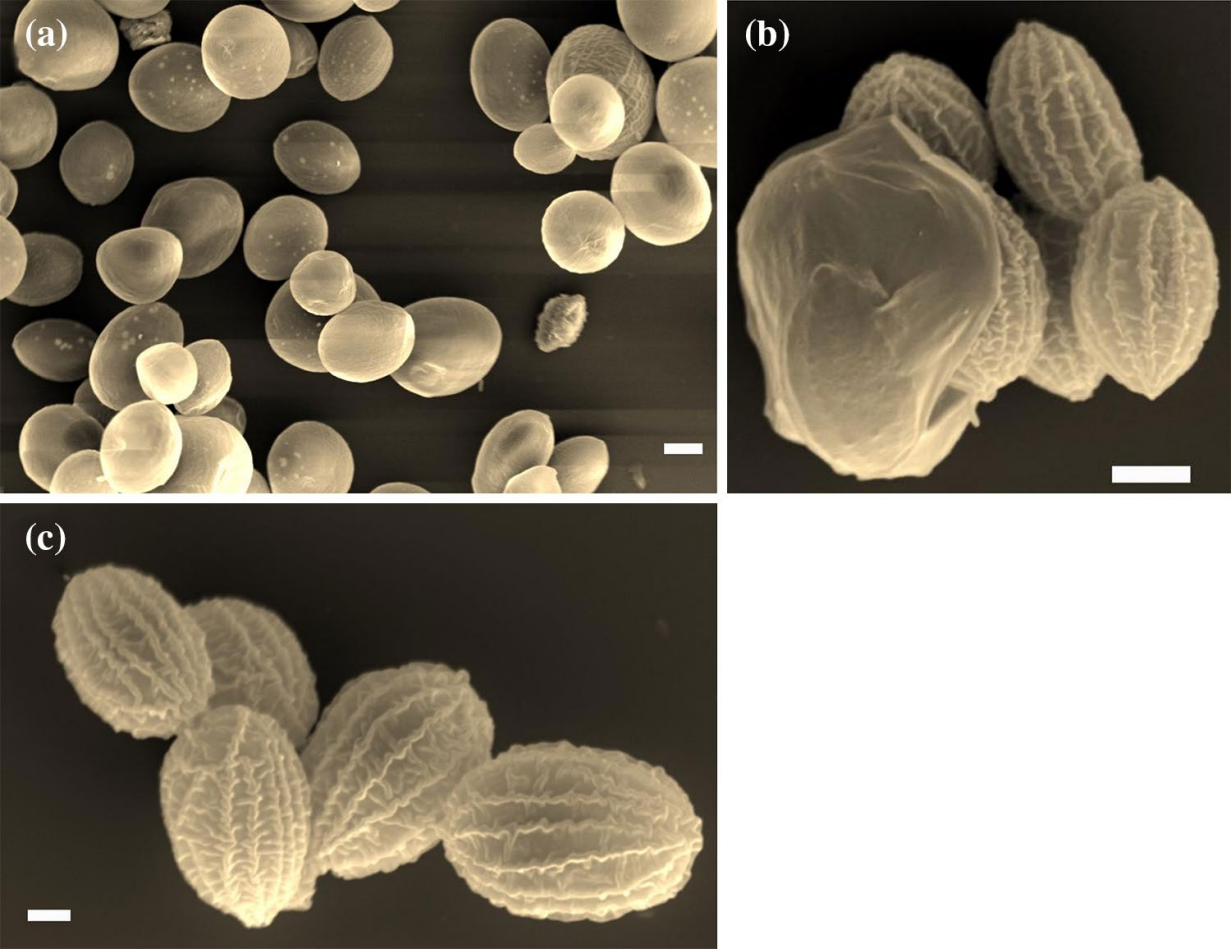
**a** Scanning electron microphotographs of a living culture after 4 days of growing. Different morphologies were observed ranging from spheroidal until ellipsoidal. Smooth ribs were visible at a surface level; **b** detail of autospores while being discharged through a rupture of the cell wall of the sporangium; **c** autospores or young vegetative cells of FGS-001, presenting a smooth pole thickening and several cell wall ribs at the surface. Scale bar for **a**, **b** and **c** = 3, 2 and 1 µm, respectively

Scanning electron microscopy was performed at different times (4–18 days) of a living culture of the strain FGS-001. Different cell morphologies were observed ranging from globose, spheroidal until ellipsoidal. As shown in the micrographs (Fig. 3), the unicellular organism presents characteristic cell wall sculptures in form of meridional ribs, although they were more clearly visible at autospores and at younger states of the cells. The number of ribs may vary accordingly, although the difference was not statistically quantified (mainly around 10–16, with a possible maximum of 20) (Fig. 3). These ribs converged at two poles of the cells, and a smooth polar thickening was formed (Fig. 3b).

Groups of autospores were discharged through a rupture of the cell wall of the sporangium which corresponded to the aggregations we observed under light microscopy (Fig. 3b, c).

### Growth characteristics

**Fig. 4:**
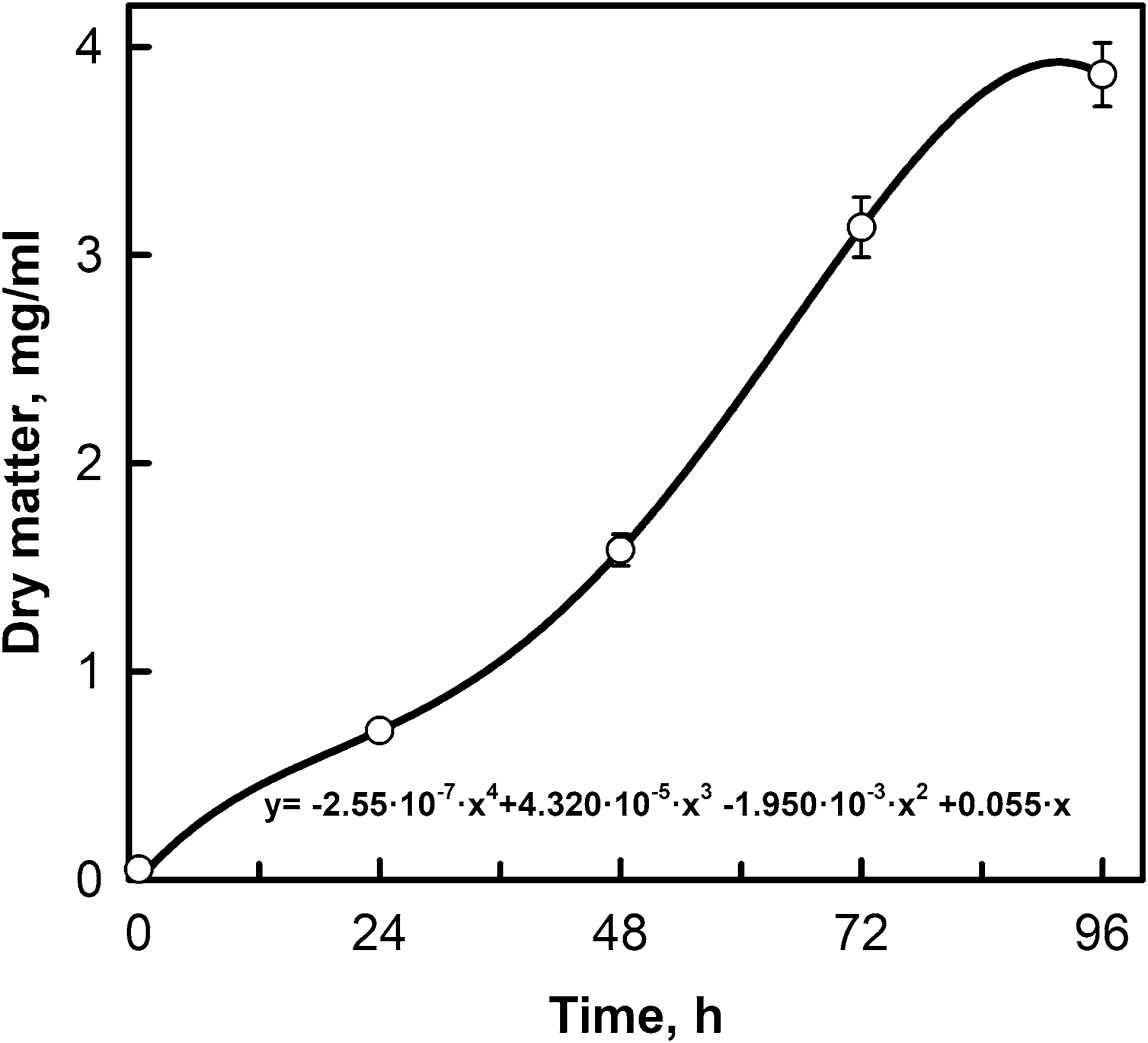
Changes in culture biomass of the green microalga *Coelastrella* sp. FGS-001 grown in an enriched media. The culture was grown in tubular bioreactors in triplicate for 4 days under constant irradiance

*Coelastrella* sp. FGS-001 was cultured in a nutrient rich media at 175 µmol/m^2^/s and 20.0 ± 2 °C temperature for 10 days to observe the growth pattern in batch culture. The alga grew well in the temperature range of 15–20 °C (data not shown). Dry matter was measured (Fig. 4). The stationary phase was observed from day 8 onwards, probably due nutrient limitation in the media.

### Phylogenetic associations

**Fig. 5:**
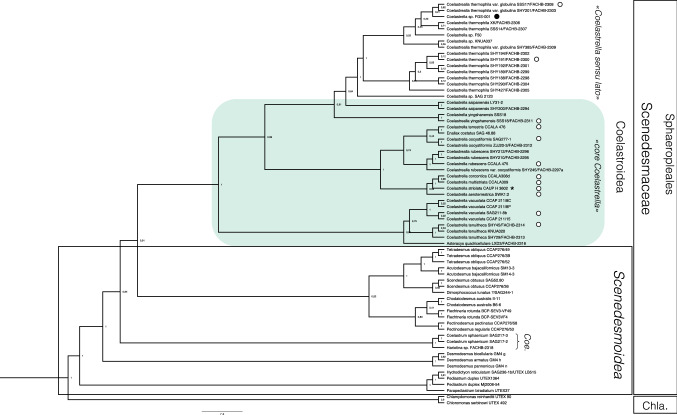
Maximum clade credibility tree of Scenedesmaceae inferred from 18S + ITS rDNA region. Numbers at nodes denote posterior probabilities (PP). Chlamydomonadales (Chla) was used as outgroup. Type species (holotype) of the genus *Coelastrella* is depicted with an asterisk. Original strains for species description are depicted with an empty circle. Strain FGS-001 presents a full circle. Most original strains for *Coelastrella* grouped together in the “core *Coelastrella*” as a sister group of the “*Coelastrella *sensu lato”, both placed into de Coelastroidea subfamily of Scenedesmaceae. Coelastroidea forms a sister group with Scenedesmoidea, except of colony-forming members of the subfamily Coelastroidea (Coe), which were included into the latter group

The nuclear 18S rDNA gene and ITS regions of the isolate FGS-001 was sequenced and compared to those of similar species in GenBank. As shown in Fig. 5, this new strain was grouped into one clade with other *Coelastrella* species and confirmed that the subfamily Coelastroideae are included in the monophyletic family Scenedesmaceae. We named the strain tentatively *Coelastrella* sp. FGS-001.

*Coelastrella striolata* (type species of the genus) and most of the available original culture strains seems to form a “core *Coelastrella*” group (Fig. 5). Our strain FGS-001 was placed within the “*Coelastrella *sensu lato” group, a sister group from the core *Coelastrella* group.

### Pigment analysis

**Fig. 6:**
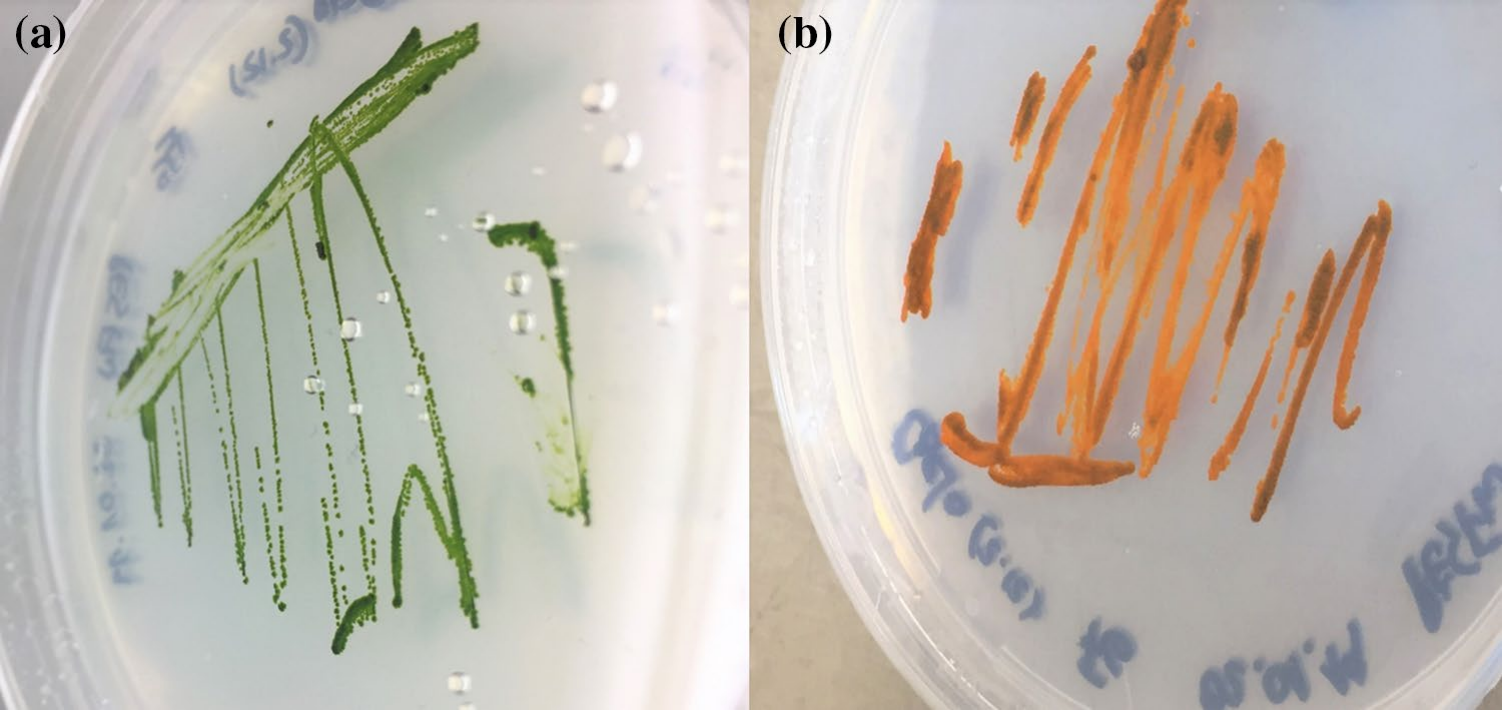
A plate culture before (**a**) and after (**b**) the stationary phase, showing the drastic change of color of the strain

At the exponential phase, the main pigments of the strain FGS-001 were chlorophyll a and b, represented by a strong green color (Fig. 1b). After a few weeks at the stationary phase and without replacement of new nutrient media, the culture turned into yellowish-green until reddish-orange. This can be easily demonstrated on an agar plate at the exponential phase (Fig. 6a), and after a few weeks later of growth at the stationary phase (Fig. 6b). In the latter biomass, we were able to detect the presence of neoxanthin, pheophytin a, astaxanthin, canthaxanthin, lutein, an unknown carotenoid, and violaxanthin as the responsible pigments for this orange color (Fig. 6b, Table 2); although pigments were not quantified.

### Fatty acid results

The fatty acid (FA) composition of freeze-dry biomass of the microalgal strain was analyzed using a gas chromatograph-mass spectrometer. We were able to identify 17 fatty acids after 8 and 18 days of cultivation, which were composed of saturated and unsaturated FAs with 10 to 20 carbon atoms. The FA composition is represented in Table 3.

**Fig. 7:**
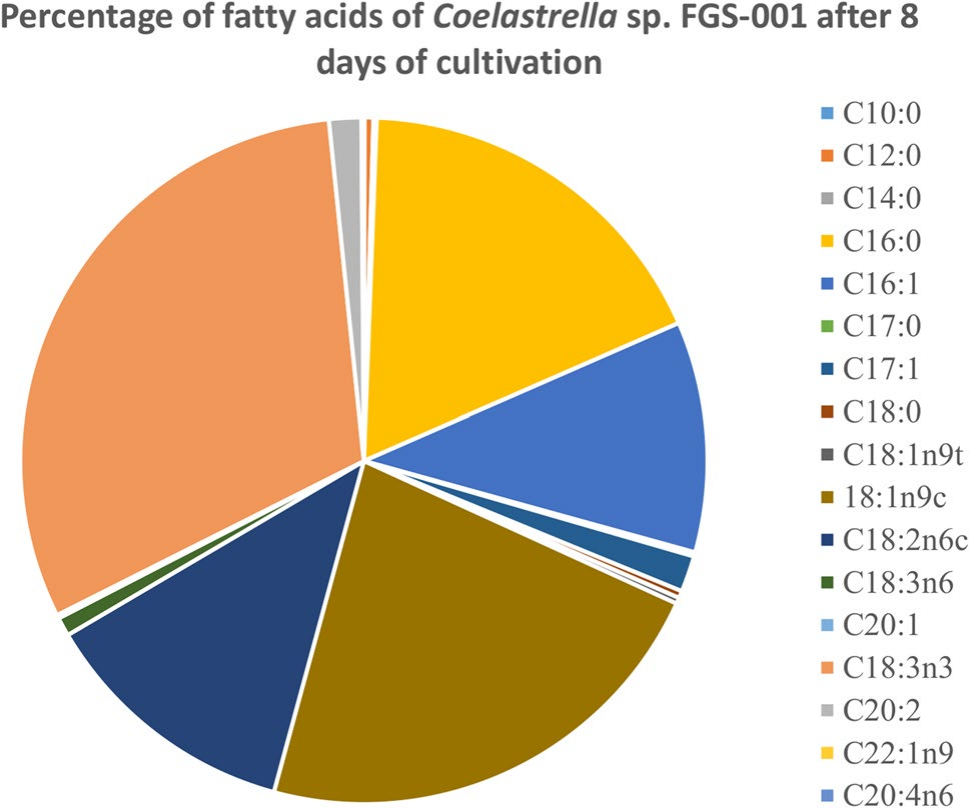
Detailed percentage distribution on the fatty acid profile of *Coelastrella* sp. FGS-001 after 8 days of cultivation. Linolenic acid (C18:3 n3) corresponded to the main fatty acid (with 30.8%), followed by oleic acid, palmitic acid and linoleic acid

The major FAs in *Coelastrella* sp. FGS-001 (see Fig. 7) were C18:3 n3 (which ranged from 30.79 to 33.45% for 8 and 18 days cultivation, respectively); C18:1 n9c (22.40% to 23.33%); C16:0 (17.84% to 18.27%), C18:2 n6c (12.31% to 7.3%), and C16:1 (10.86% to 11.54%). It was found that the total amount of FAs in the algae cells was 90.2 to 96.9 mg/g after first and second sampling, from which 27.8 ± 6.5 to 32.4 ± 4.8 mg/g corresponded to linolenic acid, the main fatty acid on the microalga (Table 3, Fig. 7).

The average percentages of total polyunsaturated fatty acids (PUFAs) were the highest (45.5% to 44.1%), in comparison with total saturated fatty acids (SFAs; 18.9% to 19.3%) and total monounsaturated fatty acids (MUFAs; 35.5% to 36.6%) (Fig. 7, after 8 days cultivation).

Slight differences in the FA percentage were observed among 8 and 18 days of cultivation (Table 3). A slight increase was observed on the FAs C16:0; C16:1; C17:0; C18:1 n9c; C18:3 n6; C20:1; C18:3 n3; C20:2, and C22:1 n9, although less than 3%. C18:2 n6c was reduced in 5% after 18 days, and it was the most notorious change in the FA profile after 10 days difference.

## Discussion

In the present study, we described a terrestrial green microalga isolated at Ås, Norway. The strain corresponded to a coccoid chlorophyte. Morphological characteristics by light, fluorescence and electron microscopy (using TEM and SEM), in conjunction with sequences of 18S rDNA and ITS region, were used to identify the strain. The characteristics agree with those of the genus *Coelastrella* defined by Chodat (Table 1), with a sister relationship with *Coelastrella thermophila* var. *globulina* recently described by Wang et al. (2019b). A list of all the accepted species of *Coelastrella* and their morphological features is displayed at Supplement Table 1.

**Table 1:** Comparison of diacritical morphological features among “similar” genera of green coccoid microalgae, based on data recorded in AlgaeBase (Guiry and Guiry 2020) and original type strain species descriptions (Fas = flagged as accepted taxonomically by literature)

Genus	Organization	Cell walls (CW)	Chloroplast (CH), pyrenoid (Py)	Asexual reproduction	Phylogeny
*Coelastrella* Chodat 1922, (16 Fas)*	solitary, temporary aggregated	16–40 ribs, with AM	1CH, parietal, cup-shaped; 1Py	2–16 autospores	SphaO; SceF; CoeS *polyphyletic
*Graesiella* Kalina and PunČochářová 1987, (1 Fas)	solitary	smooth CW, fine network of ribbing	1CH, parietal; 1Py	2–8–(16) autospores	ChlaO incertae sedis
*Scotiella* (Chodat) Fritsch 1912, (9 Fas)	solitary	6 defined longitudinal ribs	–	–	(ChlaO; ChloF) *many now as Chloromonas*
*Scotiellopsis* Vinatzer 1975, (1 Fas)*	solitary, temporarily 2–4–(8) celled colonies	meridional ribs pole to pole, with AM	1CH, parietal, 1Py	2(–16) autospores	SphaO; SceF; CoeS *soon replaced?
*Asterarcys* Comas Gonzales 1981, (1 Fas)	2–4–8 celled coenobia embedded in mucilage	thin and smooth CW, with AM	1CH, parietal cup-shaped; 1Py	4–8 autospores	SphaO; SceF; CoeS
*Hariotina* Dangeard 1889, (2 Fas)	4 celled coenobia	–	–	Daughter colonies	SphaO; SceF; CoeS
*Dimorphococcus* Braun 1855, (3 Fas)	colonies of 4-celled coenobia with 16- or more- celled syncoenobia	Smooth CW	1CH, parietal; 1-3Py	4 autospores	SphaO; SceF; CoeS
*Coelastrum* Nägeli 1849, (30 Fas)	4, 8, 16, 32 or 64 (-128) celled coenobia	smooth and wrinkled, specialized wall plaques	1CH, parietal; 1Py	daughter colonies	SphaO; SceF; CoeS
*Enallax* Pascher 1943, (2 Fas)*	2–4-8 celled coenobia not embedded in mucilage	3–6 longitudinal ribs, pole to pole	1CH, parietal; 1Py	2–8 autospores ()	SphaO; SceF; SceS *valid genus?
*Ettlia* Komárek 1989, (7 Fas)*	solitary, temporary aggregated	Thin CW	1CH, cup-shaped; 1Py	4–16-(64) zoospores; aplanospores	ChlaO incertae sedis *polyphyletic
*Chlorococcum* Meneghini 1842, (47 Fas)*	solitary, temporary aggregated	Smooth CW	1CH, parietal, cup-shaped; 1Py or +	Motile zoospores, with 2 flagella; aplanospores	ChlaO; ChloF *polyphyletic
*Tetracystis* Brown and Bold 1964, (15 Fas)*	4 celled coenobia	–	1CH, parietal; 1Py	4–8 motile zoospores, with flagella	ChlaO; ChloF *type strain is now a *Chlorococcum*

The actual phylogeny is represented by Order Sphaeropleales (SphaO), Family Scenedesmaceae (SceF), and subfamilies Coelastroideae (CoeS), and Scenedesmoidea (SceS); and by the Order Chlamydomonadales (ChlaO), Families Chlamydomonadacea (ChlaF), and Chlorococcaceae (ChloF)

*AM* sporopollenin or other acetoresistant material

*See comments on Phylogeny

To our knowledge, *Coelastrella* has not been registered in continental Norway before; thus, there were no previous cultures available on collections of Norwegian strains. We agree that due to the scarcity of visual characters suitable for diagnostic purposes (see Table 1) there is a high possibility that many species of coccoid microalgae (including *Coelastrella* spp.) have been neglected in local environmental studies (in Norway and elsewhere). Only the last year, four new species and two new varieties have been described of *Coelastrella* (Kawasaki et al. 2019; Wang et al. 2019b). In Norway, there are few observations of other related green coccoid microalgae, including *Dimorphococcus* and *Coelastrum* (also members of the Coelastroideae), and *Chlorococcum* spp. (see Artsdatabanken 2018, https://artsdatabanken.no/; Norwegian Culture Collection of Algae, NORCCA 2018; The Culture Collection of Cryophilic Algae, CCCryo 2018; Bruteig et al. 2001). Moreover, Kol (1963) described the species *Scotiella norvegica* from red snow of Finse, Hordaland County (west Norway), although a phylogenetic position of this species is lacking. Many fusiform snow algae inhabiting polar areas and high alpine zones were initially believed to be a member of this genus, but further studies have pointed several of the *Scotiella* 'species' as zygospores of chlamydomonad-like snow algae (Procházková et al. 2018; Remias et al. 2018; Table 1).

Further north, in the Svalbard archipelago, Kim et al. (2008) investigated snow algae from northwestern Spitsbergen and reported a *Scotiellopsis* sp. At the same area, another group of researchers collected *Scotiella* sp. from supraglacial sediments (Stibal et al. 2006), and *Scotiella norvegica* and *Scotiellopsis terrestris* from wet soil and moss samples (Matuła et al. 2007). Nevertheless, over the last years, several species of *Scotiella* and *Scotiellopsis* were transferred to other genera including *Coelastrella* (Kaufnerová and Eliáš 2013). Recently, in Svalbard, *Coelastrella aeroterrestrica, Coelastrella rubescens, Coelastrella cf. rubescens,* and a *Coelastrella* sp., were identified by Borchhardt et al. (2017). However, apparently only strains of *Chloromonas nivalis* (ex *Scotiella antarctica* Fritsch, ex *Scotiella nivalis* (Chodat) Fritsch, ex *Scotiella cryophila* Chodat) are available from Svalbard in culture collections (see The Culture Collection of Cryophilic Algae, CCCryo 2018). This makes us conclude that there are no previous cultures available on collections of Norwegian strains.

### Strain morphological identification

The strain FGS-001 grew solitary, especially under aeriation, and only small groups of cells were temporary aggregated, probably after autospores liberation. Without any aeration, the strain tended to form biofilm. Cells were also not embedded in mucilage. The family Scenedesmaceae contains numerous coenobial species of *Desmodesmus*, *Neodesmus*, and especially *Scenedesmus*, although some representatives of the latter genus are only known in solitary coccoid form. Other genera in this subfamily are either coenobial (e.g., *Coelastrum*, *Hariotina*) or solitary (e.g., *Coelastrella*, *Scotiellopsis*) (Fučíková et al. 2014). Thus, cell organization is a relevant taxonomic character for differentiation among Coelastroidea genera e.g., mainly solitary genera, coenobial genera, and mucilage producing genera (Table 1). Nevertheless, using light microscopy observations of living cells was almost impossible to assign any genus to the strain (Fig. 1). Furthermore, with fluorescence techniques was it possible to determine that cells are uninucleate (Fig. 1d), and that bigger structures (autosporangia) contained several cells inside (data not shown).

The apparent lack of cells with flagella, or any other type of zoospores (or potentially motile cells) in asexual reproduction, the presence of stigma, are definitive characters for differentiating the strain from Chlamydomonadales (Fig. 1, Table 1). As observed by other authors and for several *Coelastrella* species, the strain produced secondary carotenoids under stress or in older cultures (Fig. 6; PunČochářová and Kalina 1981; Abe et al. 2007; Hu et al. 2013; Kawasaki et al. 2019).

Scanning electron microscopy was determinant to discern the details of the cell wall surface. In this case, we could clearly see the presence of longitudinal ribs, which cover from pole to pole. They were especially defined at early stages of the cells (Fig. 3c), but they still could be observed in older and bigger cells (Fig. 3a). A similar cell wall surface has been registered by SEM for different *Coelastrella* species around the world, i.e. *C. astaxanthina* (Kawasaki et al. 2019), *C. ellipsoidea* and *C. multistriata* var. *grandicosta* (Gopalakrishnan et al. 2014); *C. terrestris* (PunČochářová and Kalina 1981; Gärtner and Ingolić 1993; Tschaikner et al. 2007a, b; Kaufnerová and Eliáš 2013; Xiao et al. 2017); *C. aeroterrestrica* (Uzunov et al. 2008); *C. oocystiformis* (PunČochářová and Kalina 1981), *C. rubescens* (PunČochářová and Kalina 1981; Kaufnerová and Eliáš 2013); *C. saipanensis* strain FACHB-2138 (Wang et al. 2019a), *C. thermophila* (Wang et al. 2019b), *C. vacuolata* (Hanagata 2001); *C. multistriata* var. *multistriata* (PunČochářová and Kalina 1981; Kalina and PunČochářová 1987; Hanagata et al. 1996); *C. striolata* (Kalina and PunČochářová 1987), *C. yingshanensis* (Wang et al. 2019b), *Coelastrella* sp. F50 (Hu et al. 2013); and *Coelastrella* sp. YC001 (Lee et al. 2016). The presence of longitudinal ribs is a clear character that helps to distinguish among genera; small thickenings at the poles, and a citriforme until globose morphology, are also considered relevant taxonomic characters (Table 1). For comparison with other close related species, i.e. other Scendesmaceae, see Kalina and PunČochářová (1987); Hanagata et al. (1996); Comas and Krienitz 1997; An et al. (1999); Hegewald et al. (2010); Eliáš et al. (2010); and Gopalakrishnan et al. (2014); or for Chlamydomonadales see Procházková et al. (2018) and Remias et al. (2018).

Using TEM, Gärtner and Ingolić (1993) and later Tschaikner et al. (2007b) showed a very similar liberation of daughter cells/autospores from an autosporangium from *C. terrestris* (strain N29 and strain SWK3:53 respectively, ex *S. terrestris*). Similar observations were made by PunČochářová and Kalina (1981) and Kaufnerová and Eliáš (2013) in *C. rubescens* CCALA 475, Uzunov et al. (2008) in *C. aeroterrestrica*, and Kawasaki et al. (2019) in *C. astaxanthina* and *Coelastrella* sp. SAG 2123. The number of daughter cells/autospores released by the sporangium is considered of taxonomic relevance as well, among these microalgae (Table 1). We observed that apparently the strain FGS-001 liberated 2 to 6 autospores (Figs. 2c and 3b).

Transmission electron microscopy was useful to observe the inner structure and distribution of organelles on the cells. The characteristic complex cell wall was also seen with this method (Fig. 2e), a taxonomic characteristic of the genus (Table 1). The different ribs types were also observed with TEM (Fig. 2d). The chloroplast form position and the clear pyrenoid complex were recorded too and appears to be as described for *Coelastrella* (Fig. 2b). For comparison with related or “similar” green coccoid microalgae investigated with TEM see Pickett-Heaps and Staehelin (1975), Gopalakrishnan et al. (2014) or Shebanova et al. (2017) for *Desmodesmus* and *Scenedesmus* spp., Chihara et al. (1994), Hu et al. (1998) and Feng et al. (2016) for *Chlorococcum*, Cardon et al. (2018) for *Enallax costatus* and *Acutodesmus* spp., Eliáš et al. (2010) for *Hylodesmus*, Matsuzaki et al. (2018), and Procházková et al. (2018), and Remias et al. (2018) for *Chloromonas* spp.

Pickett-Heaps and Staehelin (1975) showed the presence of other structures on the cell wall, like terminal spines, which is characteristic in many *Scenedesmus* species. Interestingly, Miller (1978) presented TEM microphotographs of zoospores of *Chlorococcum oleofaciens* (Utex 105), which is very useful for comparison with our strain. The authors showed the chlamydomonal-like structures, i.e. cell wall surface, stigma, flagella and a posterior nucleus, which are absent in our isolate.

### Strain phylogeny

A faster and more accurate alternative for species identification and phylogenetic relationships among coccoid algal species is provided by DNA sequence comparisons (Škaloud et al. 2016). In general, our phylogenetic analysis of 18S rDNA and ITS region confirmed that the subfamily Coelastroideae are included in the monophyletic family Scenedesmaceae, although a more detailed analyses showed that the *Coelastrella* taxa belong to several different lineages within this family (Fig. 5). Our analyses did not clearly define a unique lineage for the *Coelastrella* taxa but add a new strain for such future elucidation. Similar conclusions were made by previous studies (see Hegewald et al. 2010; Kaufnerová and Eliáš 2013; Lee et al. 2016; Wang et al. 2019a).

Based on the 18S rDNA and ITS sequence phylogenetic tree phylogeny, we placed our strain into the order Sphaeropleales, as a sister order of the Chlamydomonadales, as observed previously (Hodač 2015; Watanabe and Lewis 2017; Fig. 5). Several morphological, ultrastructural and reproductive characteristics are shared (or appear to be) between these coccoid green microalgae (Table 1), therefore misidentification has been a problem for further phylogenetic studies. Several sequences available online carried misleading names and complicates the analysis of the group’s phylogeny (see Kaufnerová and Eliáš 2013; Lee et al. 2016). As mentioned, certain genera, as *Scotiella*, were found to be resistant stages (which presented a similar morphology to Coelastroideae: non-motile cysts with longitudinal ribs on cell surface) of other green algae as Chlamydomonadales i.e. *Chloromonas* spp. (Procházková et al. 2018; Remias et al. 2018). The genus are retained only for those species whose reproduction remain unknown (Hanagata 1998). Other genera have been separated from species (i.e. *Scotiellopsis*) by transferring them to other genera, based on new ultrastructural or molecular methods; remaining only few (old) species with unknown, too difficult to get, or none, biological material for further comparisons. Hence the importance of strain deposition on culture collections.

Similarly, as in Kaufnerová and Eliáš (2013), the type species of *Coelastrella* (*C. striolata*) and most of the available original (type culture) strains used for the description of several species of *Coelastrella* seems to form a “core *Coelastrella*” group (Fig. 5). Our strain FGS-001 grouped clearly into the subfamily Coelastroidea (family Scenedesmaceae) as well, specifically into the “*Coelastrella *sensu lato” group, a sister group from the core *Coelastrella*.

Further molecular phylogenetic investigations have raised concerns regarding the polyphyletic nature of some of those genera and further modifications are expected to happen as is the case of *Chlorococcum* (Kawasaki et al. 2015; Feng et al. 2016), *Coelastrella* (Kaufnerová and Eliáš 2013; Wang et al. 2019a), *Coelastrum* (Hegewald et al. 2010), and *Ettlia* (Pegg et al. 2015). Using 18 s rDNA sequences, Kaufnerová and Eliáš (2013), and later Wang et al. (2019a) demonstrated that the genus *Coelastrella *sensu lato is paraphyletic as species currently attributed to the genera *Asterarcys, Scenedesmus* or *Ettlia* are nested among taxa nominally representing *Coelastrella*. Clearly internal relationships among *Coelastrella* species are not solved yet and denser taxon sampling with more molecular markers is required to elucidate the classification of the strains (Lee et al. 2016). As mentioned by Wang et al. (2019b), it seems that 18S rDNA was too conserved to be used as a species-specific marker in this clade. We should clarify that a few original strains lacked available 18S sequences as *Coelastrella compacta*, *C. levicostata*, and *C. coelastroides* (see Supplement Table 1), which could also help to elucidate the phylogenetic relationships among these taxa. We contribute with a new strain isolated from northern Europe, and placed in a culture collection, which can help for further elucidation.

Recently, Wang et al. (2019a) studied the chloroplast genome sequence (cpDNA) of the Chinese strain *Coelastrella saipanensis* FACHB-2138. This is the first report on the cpDNA structure of the genus *Coelastrella*. Chloroplast genes often provide stronger phylogenetic signals, however, in the case of Scenedesmaceae, only very few chloroplast genomes are available.

### Pigment analysis

Fresh cultures of the strain FGS-001 presented a strong green color due chlorophyll a and b, as main pigments, but after a few weeks under normal conditions, they turned into yellowish-green until reddish-orange after the stationary phase (Fig. 6, Table 2). HPLC–DAD for maximal extinction determination and UPLC-MS for mass detection, were used to determine those carotenoids. We detected the presence of neoxanthin, pheophytin a, astaxanthin, canthaxanthin, lutein, unknown carotenoid, and violaxanthin. A similar profile has been observed in several strains of *Coelastrella* as discussed below.

**Table 2:** Pigment composition of biomass of *Coelastrella* sp. FGS-001 after the stationary phase

Name	Comment
Neoxanthin	–
Pheophytin a	–
Astaxanthin	Both isomers
Canthaxanthin	Both isomers
Lutein	Both isomers
Unknown carotenoid	(m/z) appr. 468
Violaxanthin	–
Chlorophyll a	Major chlorophyll
Chlorophyll b	–

Abe et al. (2007) studied the pigment composition of a Japanese strain of *Coelastrella striolata* var. *multistriata*, before and after the stationary phase. The authors demonstrated the shift in pigment concentrations from chlorophyll (Chl a, b) dominance, until a carotenoid dominance (β-carotene, canthaxanthin and astaxanthin) after the stationary phase.

Hu et al. (2013) isolated a Chinese thermotolerant strain, *Coelastrella* sp. M-60 (Hu 2012), which under environmental stress (i.e., salt stress) produced astaxanthin (1.8% dw), β-carotene (1.4%), lutein (0.7%), canthaxanthin (0.18%), and adonirubin. Interestingly, 18S rDNA phylogeny grouped this strain with FGS-001 close to each other (Fig. 5). Recently, Karpagam et al. (2018) studied more in detail the carotenoid biosynthesis pathway in this strain.

Minyuk et al. (2017) studied the stress-induced secondary carotenogenesis in *Coelastrella rubescens* CCALA 475 and observed the shifts in the production of pigments (neoxanthin, violaxanthin, anteraxanthin, lutein, zeaxanthin, canthaxanthin, astaxanthin, adonixanthin, α- and β-carotene) under different culture treatments (N, P, sodium acetate, and CO_2_).

On one hand, the pigment profile of microalgae have been considered as a relevant taxonomical character in microalgae (Serive et al. 2017), which supported in this case the affiliation of our strain into *Coelastrella* spp. (Fig. 5) On the other hand, carotenoids are bioactive compounds having characteristic antioxidant, antimicrobial, antiviral, anti-tumoral, anti-inflammatory and anti-allergy effects, which give rise to health benefits (Karpagam et al. 2018).

Habitats where species of *Coelastrella* grow (with dehydration, temperature stress, salt stress, and high-light exposure) favors the production of pigments and fatty acids. *Coelastrella* species usually show strong survivability under such extreme photooxidative stresses; and therefore, they are considered good candidate species for large-scale production of natural pigments and biofuels (Wang et al. 2019b).

### Fatty acid analysis

Gas chromatography analyses of the FAME-derivatives of the strain FGS-001 showed 17 fatty acids ranging from C10 until C20. The main fatty acids contained 16 and 18 carbon atoms and were identified as linolenic acid, oleic acid and palmitic acid, and palmitoleic acid (Fig. 7, Table 3).

**Table 3:** Detailed fatty acid composition of the isolated microalga FGS-001 under cultivation on day 8 and 18 (in mg/g; mean ± SD; n = 3)

Fatty acid	Name	8 days	18 days
C10:0	Capric acid	0.027 ± 0.01	0.033 ± 0.01
C12:0	Lauric acid	0.370 ± 0.02	0.359 ± 0.04
C14:0	Myristic acid	0.140 ± 0.01	0.156 ± 0.01
C16:0	Palmitic acid	16.083 ± 0.75	17.700 ± 2.60
C16:1	Palmitoleic acid	9.790 ± 1.08	11.183 ± 1.30
C17:0	Heptadecanoic acid	0.177 ± 0.02	0.214 ± 0.05
C17:1	*Cis*-10-heptadecanoic acid	1.539 ± 0.30	1.275 ± 0.13
C18:0	Stearic acid	0.305 ± 0.05	0.242 ± 0.03
C18:1n9t	Elaidic acid (*trans*)	0.259 ± 0.14	0.107 ± 0.02
C18:1n9c	Oleic acid (*cis*)	20.191 ± 2.15	22.608 ± 2.74
C18:2n6c	Linoleic acid (*cis*)	11.102 ± 3.94	7.127 ± 0.50
C18:3n6	Ƴ-Linolenic acid	0.796 ± 0.08	0.894 ± 0.08
C20:1	Cis-11-Eicosenoic acid	0.149 ± 0.01	0.214 ± 0.04
C18:3n3	Linolenic acid	27.753 ± 6.55	32.406 ± 4.80
C20:2	*Cis*-11,14-Eicosadienoic acid	1.365 ± 0.60	2.280 ± 0.40
C22:1n9	Erucic acid	0.078 ± 0.01	0.074 ± 0.03
C20:4n6	Arachidonic acid	0.027 ± 0.00	0.021 ± 0.00
FA total		90.15	96.89

A diverse array of fatty acids ranging from C10 until C20 were quantified by gas chromatography analyses. The main fatty acids of the alga contained 16 and 18 carbon atoms

**Fig. 8:**
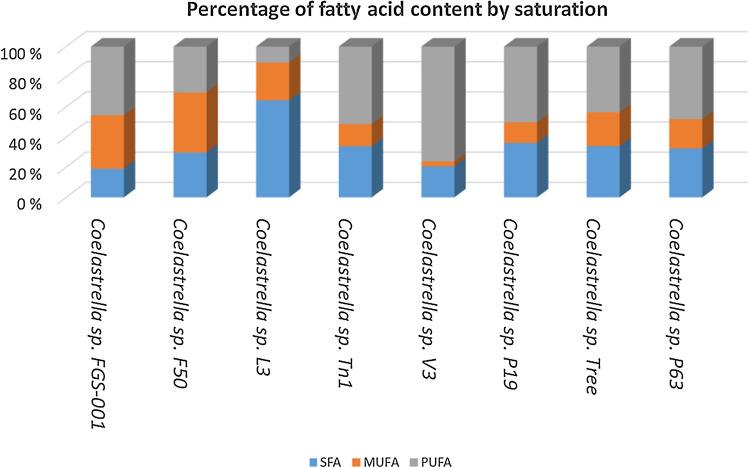
Percentage of the fatty acids by saturation (saturated (SFA), monounsaturated (MUFA) and polyunsaturated fatty acids as PUFA) present in *Coelastrella* sp. FGS-001 after 8 days of cultivation with an enriched media, and comparison with other ‘*Coelastrella*’ isolates published worldwide. Strains F50 was isolated in China (Hu et al. 2013), strain L3 was isolated in Vietnam (Thao et al. 2017), and strain Tn1 until str. P63 were isolated in India (Minhas et al. 2016). The different strains were originally grown in different nutrient media

Like other *Coealastrella* strains, relative fatty acid saturation the percentage of the fatty acids by saturation was high for MUFA and PUFA (Fig. 8). Fatty acid profiles have been considered as another taxonomical character, although we agree with Luo et al. (2016), that the lipid content and fatty acid composition are greatly affected by culturing conditions, growth period, and environmental situation (cf. Hu et al. 2013 and Minyuk et al. 2017).

Thao et al. (2017) studied a strain from Vietnam identified as *Coelastrella* sp. L3 and registered 17 fatty acids. The main fatty acids were palmitic acid, stearic acid, and oleic acid. In another species isolated from Bulgaria (*Coelastrella* sp. BGV, Dimitrova et al. (2017) identified as main fatty acids palmitic acid, oleic acid, linoleic acid and α-linolenic acid. Similarly, Abe et al. (2007) analyzed *C. striolata* var. *multistriata* isolated from Japan, and found palmitic acid, oleic acid, linolenic acid, and α-linolenic acid. Palmitic acid, α-linolenic acid and linolenic acid were the main fatty acids present in *C. rubescens* CCALA 475, a strain from Tyrol, Austria (Minyuk et al. 2017). *Coelastrella* sp. F50 isolated from China presented oleic acid, palmitic acid and linoleic acid (Hu et al. 2013); and the strain *Coelastrella* sp. QY01 from China presented palmitic acid, linoleic acid and linolenic acid as main components (Luo et al. 2016). Although the main fatty acids are shared among the mentioned *Coelastrella* strains, FGS-001 is the only one with linolenic acid as the main fatty acid, instead of palmitic acid (ranked third in our isolate).

We quantified a high percentage of polyunsaturated fatty acids (44–45%) especially linolenic acid (C18:3n3; 30–34%), therefore demonstrating interesting properties for algal biotechnology. As mentioned, the major fatty acids found in the strain were C16–C18, which are commonly found in feedstock suitable for biodiesel production (i.e. oleic acid), in terms of oxidative stability and cold flow properties (Feng et al. 2016).

The genus *Coelastrella* demonstrates interesting properties for algal biotechnology, but clearly internal relationships among species are not solved yet. We think that a thorough investigation of all those “small and neglected” microalgae groups is necessary, not only for their evolutionary, phylogenetic and ecological implications, but for biotechnology as well. In the case of the present strain FGS-001, it would be interesting to test how it behaves on a larger scale production.

## Conclusion

We investigated a coccoid green microalgae strain isolated from a terrestrial environment at Ås, Norway, and used several microscopical and molecular techniques for its identification. Coelastroideae is a subfamily in Scenedesmaceae still on the way to be clarified its phylogenetic relationships. The characteristics agree with those of the genus *Coelastrella* defined by Chodat in 1922, and the strain formed a sister group with the recently described *C. thermophila* var. *globulina*. The fatty acid analyses of the algal biomass showed a high percentage of polyunsaturated fatty acids especially from linolenic acid (30–34%). While the pigment analysis showed the presence of carotenoids like neoxanthin, pheophytin a, astaxanthin, canthaxanthin, lutein, and violaxanthin; the major fatty acids found in the strain were C16–C18, which are commonly found in feedstock suitable for biodiesel production. Therefore, the strain demonstrates interesting properties for algal biotechnology. *Coelastrella* spp. grow in habitats of high dehydration, temperature stress, salt stress, and high-light exposure, which apparently favors the production of such pigments and fatty acids.

## Electronic supplementary material

Below is the link to the electronic supplementary material.

Supplementary file1 (PDF 116 kb)—Supplement Table 1. Survey of some morphological features of Coelastrella species according to literature. (Inattachment)
