# Supplementary material for: Revision of Coelastrella (Scenedesmaceae, Chlorophyta) and first register of this green coccoid microalga for continental Norway
Source: World J Microbiol Biotechnol. 2020 Sep 11;36(10):149. doi: 10.1007/s11274-020-02897-0 (PMC7496060; doi:10.1007/s11274-020-02897-0)
Supplement: Supplementary file 1 — Supplementary file1 (PDF 116 kb)—Supplement Table 1. Survey of some morphological features of Coelastrella species according to literature. (Inattachment) [file 11274_2020_2897_MOESM1_ESM.pdf]

**Survey of some morphological features described for *Coelastrella* species according to literature.**

| Nr | spp. | Size | Place | Ribs | Body Form | Ultrastructure | Form | Daughter cells | Other: strains, sequences |
|---|---|---|---|---|---|---|---|---|---|
| **01** | *Coelastrella aeroterrestrica* Tschaikner, Gärtner & Kofler 2008 | (3,5)- 5-10 -(12)d | Tyrol, Austria, alpine | 6-16 meridional, very fine, almost invisible LM, smooth apical PT | globose to broadly ellipsoidal | 1Py, 2-3 StP, no V | solitary | 2-16 | Str. ASIB SWK 1:2, JX513879 (18S), JX513879 (ITS) |
| **02** | *Coelastrella coelastroides* Kalina 1964 | - | - | - | - | 1Py | - | - | - |
| **03** | *Coelastrella ellipsoidea* (Novis & Visnovksy) Gopalakrishnan, Novis & Visnovsky 2014 | 8-10L x 6-9w | NZ, alpine soil | smooth, 15 per cell | ellipsoidal, slight irregular | 1Py | | 2-8 | Str. LCR-CG7, KC861672 (18S) |
| **04** | *Coelastrella levicostata* Korshikov 1953 | 12-17d | Ukraine? *Sphagnum* bogs | smooth, 6-8, up to 20 longitudinal | broadly ellipsoidal to globose | | solitary | | - |
| **05** | *Coelastrella oocystiformis* (Lund) Hegewald & Hanagata 2002 | (7,5)-16-20L x(5)-12-15w | moist rocks, mountain, lake, England | 8-12(16), smooth PT, less prominent | broadly fusiform to citriform, later elliptical, SEM, TEM | 1Py, many V | solitary | | Str. SAG 277-1, Fogg, AB012848 (18S), JX513887 (ITS) |
| **06** | *Coelastrella rubescens* (Vinatzer) Kaufnerová & Eliás 2013 | 12-15 – (18)d | Tyrol, Austria, soil, calcareum | 2-6 (19) ribs, to poles, smooth PT, 2 layers CW | citriform, fusiform, bradly oval to globose, SEM, TEM | 1Py, 2 StP | solitary | 2-4-8 - (16) | Str. CCALA 475, JX513884 (18S), JX513884 (ITS) |
| **07** | *Coelastrella saipanensis* Hanagata 2001 | 4-14d | North Mariana Islands, bark | meridional, network, visible by LM | spheroidal, no polar thickening | 1Py, | solitary | 2-16 | Str. D9-1, AB055800 (18S) |
| **08** | *Coelastrella striolata* Chodat 1922 | (6).12-20-(22)L x (4,5)-9,5-18-(20,5)w | Lake area, *Sphagnum* bogs, Switzerland | 16-40 meridional, defined and other smooth, some transversal, with PT | broadly ellipsoidal to spherical, globose, SEM | 1-2Py, 2 StP, many V | solitary, rarely colonies | 2 -4-16 | Str. Kalina 1969/1, Str. E65, JX513881 (18S), JX513881 (ITS) |
| **09** | *Coelastrella terrestris* (Reisigl) Hegewald & Hanagata 2002 | (5)-14-22L x (3,5)-6-15w | Tyrol, Austria, alpine soil | 8-10-(12) meridional and defined, often fine transversal ribs, with PT, 2 layers CW | broadly ellipsoidal to citriformis, SEM, TEM | 1Py, 2 StP many V | solitary | 2-4-8-(16) | Str. CCALA 476, T87, N29, E89, SWK3, AB012847, JX513882 (18S), JX513882 (ITS) |

| | | | | | | | | | |
|---|---|---|---|---|---|---|---|---|---|
| **10** | *Coelastrella vacuolata* (Shihara & Krauss) Hegewald & Hanagata 2002 | 5,5-12L x 3,5-11w | | many, CW smooth on LM | broadly ellipsoidal to globose | many V | | | Str. SAG 211-8b, X56104 (18S) |
| **11** | *Coelastrella multistriata var. corcontica* ? / *C. corcontica* (Kalina & Punčochárová) Hegewald & Hanagata 2002 | 10-22L x 7-17w | Peat pools, subalpine region | many meridional ribs | globose to ellipsoidal | many small V | tetraedric colonies, rare solitary | | Str. Kalina 1967/9, CCALA 308, AB037082 (18S), JX513886 (ITS) |
| **12** | *Coelastrella striolata var. multistriata ?* (Trenkwalder) Kalina & Punčochárová 1987 | (4)-9-12-(13)L x (3)-7-11-(12)w | Soil pine wood, Tyrol, Italy | 16-40 meridional, visible at LM | oval, ellipsoidal to globose, SEM, TEM | 1Py, 2 StP, usually no V | solitary, rarely tetraedric colonies | 2-16 | Str. CCALA 309, Trenkwalder1975, T88, AB012846, JX513880 (18S), JX513880 (ITS) |
| **13** | *Coelastrella multistriata var. grandicosta ?* Gopalakrishnan, Novis & Visnovsky 2014 | 7-12L x 6-9w | NZ, Upper Canyon Creek | 9-12 distinct, longitudinal, multiple layers CW | ellipsoidal, SEM, TEM | 1Py | - | 4-8-16 | Str. LCR-CC-12-1d, KC861673 (18S) |
| **14** | *Coelastrella compacta* Skuja 1959 | 10-22L x 7-17w | Germany | smooth | ellipsoidal to oval | 1Py | rare solitary | 2-4 | - |
| **15** | *Coelastrella multistriata* var. *multistriata* | 6-10L x 3-7w | Japan, bark | 10-25 meridional and smooth, no PT | oval to citriform | 1Py, 2StP, no V | solitary | 2-16 | Str. C6-2, AB012846 (18S) |
| **16** | *Coelastrella thermophila* Wang et al. 2019 | (6)8-16(20)×(4)6-8(12) | China, wet concrete wall surface | (10)-15-22 | rice-shape to ellipsoidal | 1-2Py | - | 2-4-8-16 | Str. SHY191/FACHB-2300, MH176090 (18S) MH176114 (ITS) MH176139 (tufA) |
| **17** | *C. thermophila* var. *globulina* Wang et al. 2019 | (5)8-14(18) | China, wet concrete floor | (10)-13-20 | spherical | 1Py, many V | - | (2)-4-8-16 | Str. SSS17/FACHB-2308, MH176102 (18S) MH176128 (IST) MH176146 (tufA) |
| **18** | *Coelastrella yingshanensis* Wang et al. 2019 | (6)9-18×(5)7-12 | China | 10-14 | spindle to ellipsoidal | 1Py | solitary | 2-4-8 | Str. SSS18/FACHB-2311, MH176103 (18S) MH176129 (IST) MH176154 (tufA) |
| **19** | *Coelastrella tenuitheca* Wang et al. 2019 | 4-13-(18) | China, wet mud | none | spherical | uninucleate, many V | solitary | (2)-4-8-16-32 | Str. SHY45/FACHB-2314, MH176108 (18S) MH176122 (ITS) MH176149 (tufa) |

| | | | | | | | | | |
|---|---|---|---|---|---|---|---|---|---|
| **20** | *Coelastrella astaxanthina* Kawasaki et al. 2019 | 5-10L x 3-8w | Japan, asphalt | >27, meridional | oval, broadly ellipsoidal to spherical, obtuse polar ends, SEM | 1Py, uninucleated | solitary | - | Str. Ki-4, AB734096 (18S), AB762691 (ITS) |
| **21** | *Coelastrella* sp. FGS-001 | 8,4L x 7,1w | Norway, cyanobacteria, soil | horizontal and distinct, 10-16(20), 2 layers CW | spherical, ellipsoidal, poles, SEM, TEM | 1Py, 2 StP, V | solitary, biofilm | 2-6 | MK064224 (18S) MK040329 (ITS) |
| **22** | *Coelastrella* sp. F50 | - | China | 8-13, with PT | globose, SEM, TEM | 1Py | - | - | JQ867369 (18S), JQ867368 (ITS) |
| **23** | *Coelastrella* sp. CORE 3 | 10L x 6,7w | Yucatan peninsula | smooth meridional, with PT | globose | 1Py, 2-3 StP | - | 2-8 | KX940915 (ITS) |
| **24** | *Coelastrella* sp. P63 | - | India | none, smooth CW | spherical, SEM | - | - | - | Minhas et al. 2016 |
| **25** | *Coelastrella* sp. YC001 | 5-11d, 5-7L, 9-15L | China, pond | longitudinal, with PT, 2 layers CW | ellipsoidal to broadly globose, spheroidal, SEM, TEM | 1Py | solitary | 4-32 | Lee et al. 2016, Luo et al. 2016, KT259054 (18S) |

Size expressed in large (L), wide (w) or diameter (d). If there are registers of TEM or SEM microphotography is stated. Type of molecular sequences are also expressed. Cell wall = CW; pyrenoid = P; starch plates = StP; polar thickening = PT; strain = Str.

References: Chodat (1922), Korshikov (1953), Skuja (1959), Archibald (1973), Vinatzer (1975), Punčochářová & Kalina (1981), Kalina & Punčochárová (1987), Gärtner & Ingolić (1993), Hanagata et al. (1996), Hanagata (2001), Hegewald & Hanagata (2002), Tschaikner et al. (2007a,b), Hu (2012), Hu et al. (2013), Kaufnerová & Eliáš (2013), Kawasaki et al. (2013), Gopalakrishnan et al. (2014), Lee et al. (2016), Luo et al. (2016), Minhas et al. (2016), Ancona-Canché et al. (2017), Kawasaki et al. (2019), Wang et al. (2019a).
